# Utilization of in- and outpatient hospital care in Germany during the Covid-19 pandemic insights from the German-wide Helios hospital network

**DOI:** 10.1371/journal.pone.0249251

**Published:** 2021-03-25

**Authors:** Andreas Bollmann, Sven Hohenstein, Vincent Pellissier, Katharina Stengler, Peter Reichardt, Jörg-Peter Ritz, Holger Thiele, Michael A. Borger, Gerhard Hindricks, Andreas Meier-Hellmann, Ralf Kuhlen

**Affiliations:** 1 Heart Center Leipzig at University of Leipzig and Leipzig Heart Institute, Leipzig, Germany; 2 Department of Psychiatry, Psychotherapy and Psychosomatics, Helios Park Hospital, Leipzig, Germany; 3 Oncology Center Berlin-Buch, Helios Hospital Berlin-Buch and Berlin Cancer Institute, Berlin, Germany; 4 Department of Surgery, Helios Hospital Schwerin, Schwerin, Germany; 5 Helios Kliniken, Berlin, Germany; 6 Helios Health, Berlin, Germany; King’s College London, UNITED KINGDOM

## Abstract

**Background:**

During the early phase of the Covid-19 pandemic, reductions of hospital admissions with a focus on emergencies have been observed for several medical and surgical conditions, while trend data during later stages of the pandemic are scarce. Consequently, this study aims to provide up-to-date hospitalization trends for several conditions including cardiovascular, psychiatry, oncology and surgery cases in both the in- and outpatient setting.

**Methods and findings:**

Using claims data of 86 Helios hospitals in Germany, consecutive cases with an in- or outpatient hospital admission between March 13, 2020 (the begin of the “protection” stage of the German pandemic plan) and December 10, 2020 (end of study period) were analyzed and compared to a corresponding period covering the same weeks in 2019. Cause-specific hospitalizations were defined based on the primary discharge diagnosis according to International Statistical Classification of Diseases and Related Health Problems (ICD-10) or German procedure classification codes for cardiovascular, oncology, psychiatry and surgery cases. Cumulative hospitalization deficit was computed as the difference between the expected and observed cumulative admission number for every week in the study period, expressed as a percentage of the cumulative expected number. The expected admission number was defined as the weekly average during the control period. A total of 1,493,915 hospital admissions (723,364 during the study and 770,551 during the control period) were included. At the end of the study period, total cumulative hospitalization deficit was -10% [95% confidence interval -10; -10] for cardiovascular and -9% [-10; -9] for surgical cases, higher than -4% [-4; -3] in psychiatry and 4% [4; 4] in oncology cases. The utilization of inpatient care and subsequent hospitalization deficit was similar in trend with some variation in magnitude between cardiovascular (-12% [-13; -12]), psychiatry (-18% [-19; -17]), oncology (-7% [-8; -7]) and surgery cases (-11% [-11; -11]). Similarly, cardiovascular and surgical outpatient cases had a deficit of -5% [-6; -5] and -3% [-4; -3], respectively. This was in contrast to psychiatry (2% [1; 2]) and oncology cases (21% [20; 21]) that had a surplus in the outpatient sector. While in-hospital mortality, was higher during the Covid-19 pandemic in cardiovascular (3.9 vs. 3.5%, OR 1.10 [95% CI 1.06–1.15], *P*<0.01) and in oncology cases (4.5 vs. 4.3%, OR 1.06 [95% CI 1.01–1.11], *P*<0.01), it was similar in surgical (0.9 vs. 0.8%, OR 1.06 [95% CI 1.00–1.13], *P* = 0.07) and in psychiatry cases (0.4 vs. 0.5%, OR 1.01 [95% CI 0.78–1.31], *P*<0.95).

**Conclusions:**

There have been varying changes in care pathways and in-hospital mortality in different disciplines during the Covid-19 pandemic in Germany. Despite all the inherent and well-known limitations of claims data use, this data may be used for health care surveillance as the pandemic continues worldwide. While this study provides an up-to-date analysis of utilization of hospital care in the largest German hospital network, short- and long-term consequences are unknown and deserve further studies.

## Introduction

During the early phase of the Covid-19 pandemic, reductions of hospital admissions have been observed for several medical and surgical conditions [1–4]. In contrast, there is only scarce data on trends during later stages of the pandemic [5–7]. Moreover, the major focus of those studies was on inpatient care and emergency conditions [1–7]. This has prompted the German-wide Helios hospital network to refine a monitoring system that not only monitors hospitalization trends for several conditions, but also assesses the cumulative hospitalization deficit as the pandemic continues [5].

The aim of this study is to expand our previous work [1, 5] by providing up-to-date hospitalization trends and in-hospital outcomes (length of stay, mortality) for several conditions including cardiovascular, psychiatry, oncology and surgery cases in both the in- and outpatient setting.

## Methods

We performed a retrospective analysis of claims data of 86 Helios hospitals in Germany [8]. The Helios hospital group operates acute care hospitals, outpatient clinics, and prevention centers across Germany (https://www.helios-gesundheit.de/) and patients have free choice of healthcare providers. Consecutive cases with an in- or outpatient hospital admission between March 13, 2020 (the begin of the “protection” stage of the German pandemic plan) and December 10, 2020 (end of study period) were analyzed and compared to a corresponding period covering the same weeks in 2019 (March 15 –December 12, 2019). Cause-specific hospitalizations were defined on the basis of primary discharge diagnosis according to International Statistical Classification of Diseases and Related Health Problems [ICD-10-GM (German Modification)] codes for cardiovascular (I00.x-I99.x), oncology (C00.x-C97.x; D00.x-D48.x) or psychiatry cases (F00.x-F99.x). Surgery cases were defined according to the German procedure classification („Operationen und Prozedurenschlüssel“, OPS 5-01x-5-99x). In order to avoid double counting in cases with both a main diagnosis in one of the three disease categories and a surgical procedure, they were selected based on their main diagnosis. 1,929 cases (0.27%) with confirmed Covid-19 infection (U07.1) were excluded from this analysis (S1 Table).

Selection of the 4 categories were based on case volume as well as mix of variety of pathways with respect to disease spectrum, diagnostic (invasive and non-invasive) and treatment approaches (interventional/surgical and non-interventional).

This study was approved by the Ethics Committee at the Medical Faculty, Leipzig University (#490/20-ek). Due to the retrospective study of anonymized data informed consent was not obtained. Helios Health and Helios Hospitals have strict rules regarding data sharing because of the fact that health claims data are a sensible data source and have ethical restrictions imposed due to concerns regarding privacy. Access to anonymized data that support the findings of this study are available on request from the Leipzig Heart Institute (www.leipzig-heart.de).

Cumulative hospitalization deficit was computed as the difference between the expected and observed cumulative admission number for every week in the study period, expressed as a percentage (95% confidence interval [CI]) of the cumulative expected number. The expected admission number was defined as the weekly average during the control period. The difference between the expected and observed cumulative admission number was assessed using a χ^2^ test for the admission nadir defined as the week with the lowest admission number and the last week of the study period. The *P* values were adjusted for multiple comparisons using a Bonferroni correction.

## Results

**Fig 1:**
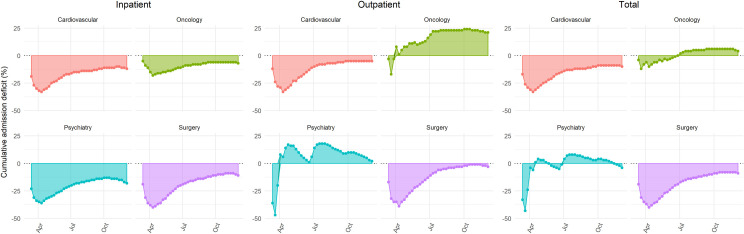
Cumulative in-, outpatient and total hospitalization case deficit for several conditions in the German-wide Helios hospital network during the Covid-19 pandemic.

In total, there were 763,067 and 895,065 inpatient, and 1,722,976 and 1,779,663 outpatient hospital cases in the 2020 study and 2019 control period. During the study period, there were 9,515 inpatient cases with SARS-Cov-2 infection. A total of 1,493,915 hospital admissions (723,364 during the study and 770,551 during the control period) were included for further analysis. There was an initial decline in weekly inpatient hospitalizations during the early phase of the study period that was followed by a recovery phase with a return to previous year inpatient case volumes but no overcompensation in all analyzed disciplines (S1 Fig). This resulted in an initial increase in the cumulative hospitalization deficit with a nadir reached in late March to mid-April, 2020 (Fig 1, Table 1).

**Table 1:** Cumulative in-, outpatient and total hospitalization case deficit for several conditions in the German-wide Helios hospital network at the nadir and in mid-December 2020 during the Covid-19 pandemic.

		Admissions until the nadir week				Admissions until the final week			
	Nadir week	Expected (n)	Observed (n)	Deficit (95% CI)	*P* Value	Expected (n)	Observed (n)	Deficit (95% CI)	*P* Value
**Cardiovascular**									
Inpatient	3 April-9 April	14,287	9,777	-32% (-33; -30)	< 0.001	139,302	122,258	-12% (-13; -12)	< 0.001
Outpatient	10 April-16 April	7,731	5,183	-33% (-35; -31)	< 0.001	60,302	57,138	-5% (-6; -5)	< 0.001
Total	10 April-16 April	25,590	17,217	-33% (-34; -32)	< 0.001	199,604	179,396	-10% (-10; -10)	< 0.001
**Oncology**									
Inpatient	10 April-16 April	11,678	9,553	-18% (-20; -17)	< 0.001	91,086	84,267	-7% (-8; -7)	< 0.001
Outpatient	20 March-26 March	3,241	2,701	-17% (-19; -14)	< 0.001	63,207	76,333	21% (20; 21)	< 0.001
Total	10 April-16 April	19,781	17,732	-10% (-11; -9)	< 0.001	154,293	160,600	4% (4; 4)	< 0.001
**Psychiatry**									
Inpatient	10 April-16 April	3,793	2,427	-36% (-39; -33)	< 0.001	29,587	24,231	-18% (-19; -17)	< 0.001
Outpatient	20 March-26 March	3,956	2,083	-47% (-50; -45)	< 0.001	77,146	78,563	2% (1; 2)	< 0.001
Total	20 March-26 March	5,473	3,129	-43% (-45; -41)	< 0.001	106,733	102,794	-4% (-4; -3)	< 0.001
**Surgery**									
Inpatient	10 April-16 April	32,304	19,340	-40% (-41; -39)	< 0.001	251,975	224,492	-11% (-11; -11)	< 0.001
Outpatient	10 April-16 April	7,429	4,527	-39% (-41; -37)	< 0.001	57,946	56,082	-3% (-4; -3)	< 0.001
Total	10 April-16 April	39,733	23,867	-40% (-41; -39)	< 0.001	309,921	280,574	-9% (-10; -9)	< 0.001

While the utilization of inpatient care and subsequent hospitalization deficit was similar in trend with some variation in magnitude between cardiovascular (-12% [-13; -12]), psychiatry (-18% [-19; -17]), oncology (-7% [-8; -7]) and surgery cases (-11% [-11; -11]), trends in outpatient care were different between disciplines during the pandemic. Cardiovascular and surgical outpatient cases had a deficit of -5% [-6; -5] and -3% [-4; -3], respectively. This was in contrast to psychiatry (2% [1; 2]) and oncology cases (21% [20; 21]) that had a surplus in the outpatient sector (Fig 1, Table 1). While in cardiovascular and surgical cases the proportion of outpatients remained stable, there was a significant increase in psychiatry and oncology (p<0.001, S2 Fig).

At the end of the study period, total cumulative hospitalization deficit was -10% [-10; -10] for cardiovascular and -9% [-10; -9] for surgical cases, higher than -4% [-4; -3] in psychiatry and 4% [4; 4] in oncology cases (Fig 1, Table 1).

Mean in-hospital length of stay was slightly shorter with less variation in cardiovascular (5.7 ± 6.6 vs. 6.0 ± 6.8 days, *P*<0.01), surgical (5.1 ± 6.6 vs. 5.2 ± 7.0 days, *P*<0.01), psychiatry (16.8 ± 20.9 vs. 17.5 ± 21.5 days, *P*<0.01) and oncology (5.7 ± 7.2 vs. 5.7 ± 7.4 days, *P*<0.01) cases during the study period.

While in-hospital mortality, was higher during the Covid-19 pandemic in cardiovascular (3.9 vs. 3.5%, OR 1.10 [95% CI 1.06–1.15], *P*<0.01) and in oncology cases (4.5 vs. 4.3%, OR 1.06 [95% CI 1.01–1.11], *P*<0.01), it was similar in surgical (0.9 vs. 0.8%, OR 1.06 [95% CI 1.00–1.13], *P* = 0.07) and in psychiatry cases (0.4 vs. 0.5%, OR 1.01 [95% CI 0.78–1.31], *P*<0.95).

## Discussion

In agreement with previous studies [1–7], a substantial inpatient hospitalization deficit has been observed for cardiovascular, psychiatry, oncology and surgical cases in Germany during the Covid-19 pandemic. On the one hand, this can be explained by a reduction in emergency admissions that has been a consistent finding in Europe and the U.S. during the early phase of the pandemic. On the other hand, non-urgent admissions and procedures had to be postponed in Germany between March 16 and end of April, 2020. Our analysis confirms and extends findings from very recent studies focusing on acute coronary syndromes [6, 7] and other cardiovascular conditions [5] during the course of the pandemic. These studies have reported a recovery phase with cases reaching almost previous year control period values. As can be appreciated from our analysis, however, there remains a substantial deficit in total cumulative hospital admissions for cardiovascular and surgical cases.

Some of this deficit may be the result of treatment avoidance during the pandemic, which may in turn result in increased out-of-hospital cardiac arrests [9] and excess mortality in the general population [10]. Delayed diagnosis or deterioration of chronic conditions followed by increased admissions, and higher morbidity and mortality is one potential scenario for which we must prepare. This is especially important, considering the increasing number of Covid-19 and other seasonal respiratory tract infections. In fact, in-hospital mortality was somewhat higher in cardiovascular and oncology cases and this finding may also contribute to the observed excess mortality in Germany that was not associated with Covid-19 death [11].

Interestingly, there were no phases of overcompensation in inpatient cases. If this is not associated with mid- and long-term increase in morbidity and mortality for certain sub-cohorts, this may suggest an overly aggressive existing model of care.

An interesting, and to the best of our knowledge a novel finding is the observation that outpatient psychiatry and oncology care was not affected or even increased. While the reasons for the latter observation are unclear, it can be speculated that patient preferences, i.e. avoidance of inpatient hospitalization, and the allocation of in-hospital resources towards Covid-19 treatment may have contributed. Whether or not this shift towards outpatient cancer care resulted in the reduction of diagnostic delays is currently unknown. The long-term effects must be closely monitored, as Covid-19 associated delays in cancer diagnosis have been suggested to substantially increase the number of avoidable cancer deaths [12].

### Limitations

Health insurance claims data is created for administrative, financial, and reimbursement purposes but not research. Nevertheless, its use has been suggested as a best fit for answering questions about health care utilization in an eligible user population [13]. Consequently, this data source may and should be used for health care surveillance as the pandemic continues worldwide.

This study focused on 4 disciplines that covered about 60% of inpatient cases in our hospitals (455,248 out of 763,067 total cases). Although they represent a variety of pathways with respect to disease spectrum and diagnostic and treatments, other disciplines were not studied which could offer additional insights.

The observed changes of in-hospital mortality during the pandemic are of interest but a detailed analysis of this observation is beyond the scope of the present study. Nevertheless, a previous study from our group that focussed on heart failure care has shown an association between increased case severity during the pandemic and in-hospital mortality [14].

## Conclusions

By analyzing a very large cohort of a hospital network representative of German hospital care and a long observation period with up-to-date data, we have identified substantial but varying changes in care pathways in different disciplines during the Covid-19 pandemic. This was associated with higher in-hospital mortality in cardiovascular and oncology care. While this study provides an up-to-date analysis of utilization of hospital care in the largest German hospital network, short- and long-term consequences are unknown and deserve further studies.

## Supporting information

S1 Fig Weekly hospital admissions. Smooth curves for weekly admission rates were fitted via Locally Weighted Scatterplot Smoothing (LOESS). Grey areas represent 95% confidence intervals.
(TIF)

S2 Fig Distribution of treatment settings. Please note the increase of outpatient treatments in psychiatry and oncology in 2020 compared to 2019.
(TIFF)

S1 Table Excluded cases due to PCR-confirmed SARS-CoV-2 infection. (DOCX)
