# Supplementary figures and images for: Utilization of in- and outpatient hospital care in Germany during the Covid-19 pandemic insights from the German-wide Helios hospital network
Source: PLoS One. 2021 Mar 25;16(3):e0249251. doi: 10.1371/journal.pone.0249251 (PMC7993839; doi:10.1371/journal.pone.0249251)

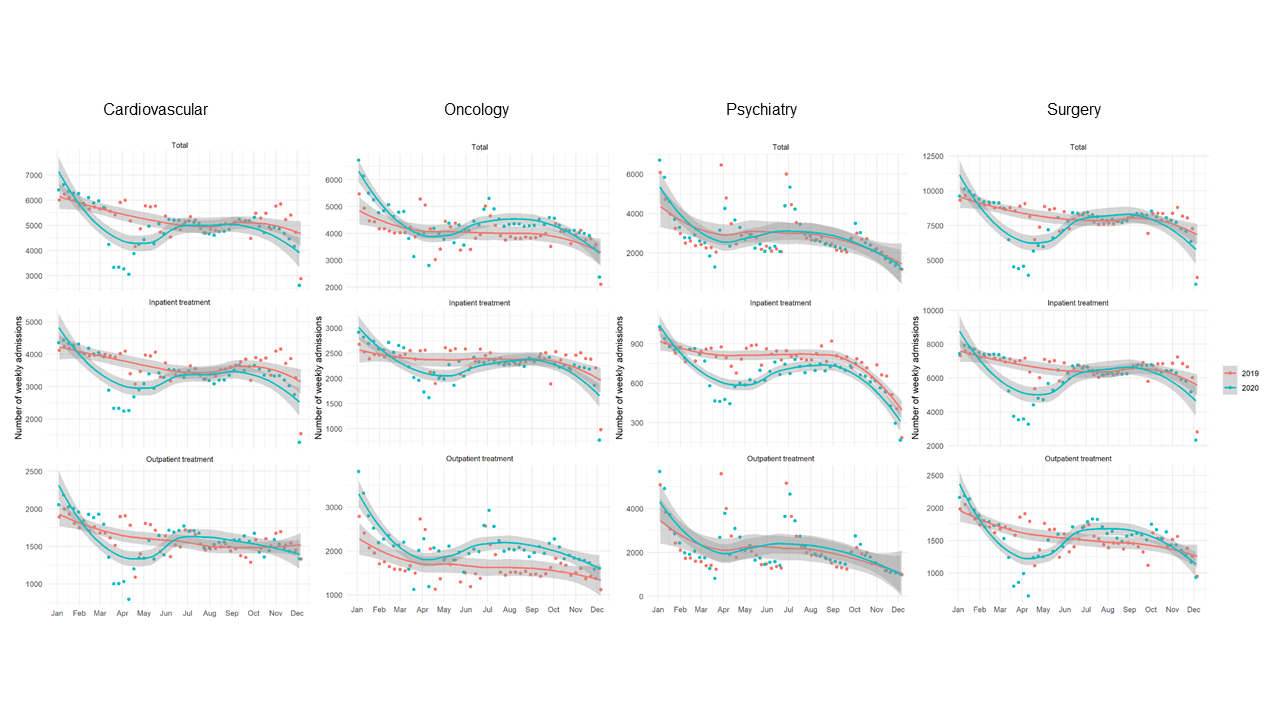

Supplement: S1 Fig — Smooth curves for weekly admission rates were fitted via Locally Weighted Scatterplot Smoothing (LOESS). Grey areas represent 95% confidence intervals. (TIF) [file pone.0249251.s001.tif]

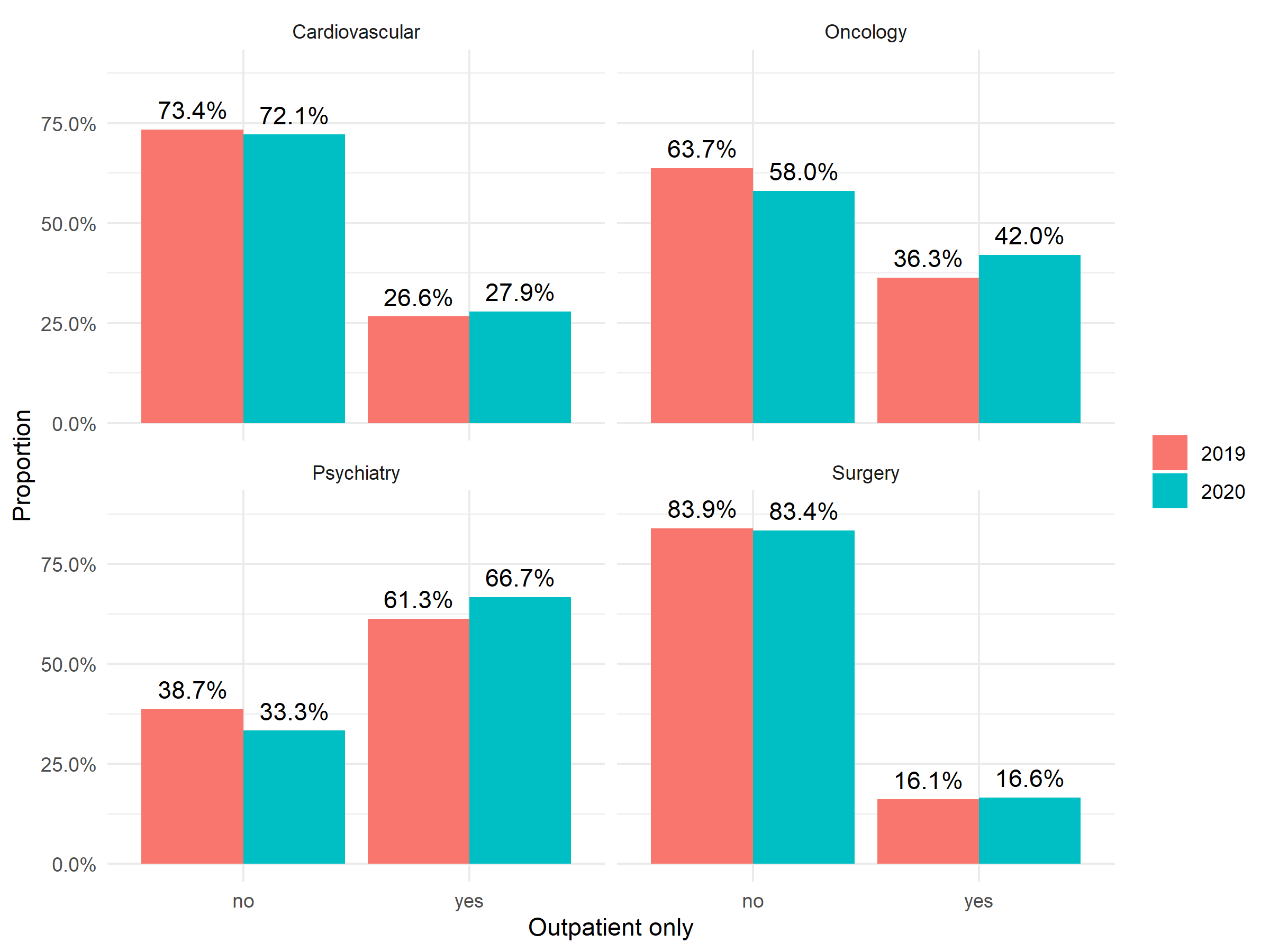

Supplement: S2 Fig — Please note the increase of outpatient treatments in psychiatry and oncology in 2020 compared to 2019. (TIFF) [file pone.0249251.s002.tiff]
