# Supplementary material for: Utilization of in- and outpatient hospital care in Germany during the Covid-19 pandemic insights from the German-wide Helios hospital network
Source: PLoS One. 2021 Mar 25;16(3):e0249251. doi: 10.1371/journal.pone.0249251 (PMC7993839; doi:10.1371/journal.pone.0249251)
Supplement: S1 Table — (DOCX) [file pone.0249251.s003.docx]

|  | **Inpatient** | **Outpatient** | **Total** |
| --- | --- | --- | --- |
| **Cardiovascular** | 495 | 79 | 574 |
| **Oncology** | 177 | 18 | 195 |
| **Psychiatry** | 81 | 28 | 109 |
| **Surgery** | 785 | 266 | 1,051 |
